# Deciphering desiccation tolerance in wild eggplant species: insights from chlorophyll fluorescence dynamics

**DOI:** 10.1186/s12870-024-05430-9

**Published:** 2024-07-25

**Authors:** Pratapsingh S. Khapte, Sushil S. Changan, Pradeep Kumar, T. H. Singh, Ajay Kumar Singh, Jagadish Rane, K. Sammi Reddy

**Affiliations:** 1 ICAR-National Institute of Abiotic Stress Management, Baramati, Maharashtra 413115 India; 2 ICAR-Central Arid Zone Research Institute, Jodhpur, Rajasthan 342003 India; 3 ICAR-Indian Institute of Horticultural Research, Bengaluru, Karnataka 560089 India; 4 ICAR-Central Institute for Arid Horticulture, Bikaner, Rajasthan 334006 India

**Keywords:** Brinjal, Dehydration tolerance, Leaf desiccation, Photosystem II efficiency, Wild species

## Abstract

**Background:**

Climate change exacerbates abiotic stresses, which are expected to intensify their impact on crop plants. Drought, the most prevalent abiotic stress, significantly affects agricultural production worldwide. Improving eggplant varieties to withstand abiotic stress is vital due to rising drought from climate change. Despite the diversity of wild eggplant species that thrive under harsh conditions, the understanding of their drought tolerance mechanisms remains limited. In the present study, we used chlorophyll fluorescence (ChlaF) imaging, which reveals a plant’s photosynthetic health, to investigate desiccation tolerance in eggplant and its wild relatives. Conventional fluorescence measurements lack spatial heterogeneity, whereas ChlaF imaging offers comprehensive insights into plant responses to environmental stresses. Hence, employing noninvasive imaging techniques is essential for understanding this heterogeneity.

**Results:**

Desiccation significantly reduced the leaf tissue moisture content (TMC) across species. ChlaF and TMC displayed greater photosystem II (PSII) efficiency after 54 h of desiccation in *S. macrocarpum*, *S. torvum*, and *S. indicum*, with *S. macrocarpum* demonstrating superior efficiency due to sustained fluorescence. PSII functions declined gradually in *S. macrocarpum* and *S. torvum*, unlike those in other species, which exhibited abrupt declines after 54 h of desiccation. However, after 54 h, PSII efficiency remained above 50% of its initial quantum yield in *S. macrocarpum* at 35% leaf RWC (relative water content), while *S. torvum* and *S. indicum* displayed 50% decreases at 31% and 33% RWC, respectively. Conversely, the susceptible species *S. gilo* and *S. sisymbriifolium* exhibited a 50% reduction in PSII function at an early stage of 50% RWC, whereas in *S. melongena*, this reduction occurred at 40% RWC.

**Conclusion:**

Overall, our study revealed notably greater leaf desiccation tolerance, especially in *S. macrocarpum, S. torvum*, and *S. indicum*, attributed to sustained PSII efficiency at low TMC levels, indicating that these species are promising sources of drought tolerance.

**Supplementary Information:**

The online version contains supplementary material available at 10.1186/s12870-024-05430-9.

## Introduction

Abiotic stresses are exacerbated by climate change, and their effects on crop plants are expected to be amplified in this century. Among abiotic stresses, drought is the most prevalent stress affecting agricultural production worldwide. Eggplant (*Solanum melongena* L.) is an important vegetable in the diet of people, particularly in tropical and subtropical regions of the World [1]. Eggplant fruits are rich in bioactive compounds and have oxygen radical absorbance capacity [2, 3]. However, due to climate change, several abiotic stressors have severely hampered eggplant crop production [4]. Therefore, there is a pressing need to identify stress-tolerant genotypes and germplasms through screening, particularly crop wild relatives, while developing an understanding of the mechanism underlying abiotic stress tolerance [5, 6]. In arid and semiarid regions of the World, due to low and erratic precipitation and poor water retention capacity of soils, plants experience transient differences in soil moisture during their production cycle. This, coupled with low relative humidity, leads to a rise in vapour pressure deficit, ultimately triggering desiccation of leaves. Desiccation in plants is one of the extremely harsh physical conditions an organism can experience, and it can harm its growth, development, and metabolism [7].

India is the primary center of eggplant diversity and has a large number of wild relatives of eggplant species found in its natural habitat [8]. The categorization of the wild species of eggplant is determined by their capacity for crossbreeding with cultivated varieties, following the gene pool concept. This classification includes primary (GP1), secondary (GP2), and tertiary gene pools (GP3) as outlined by Harlan and de Wet [9]. These wild species can be potential sources of abiotic tolerance, as highlighted by Meyer et al. [10] and Knapp et al. [8]. Therefore, it is necessary to gain a better understanding of the mechanisms underlying abiotic stress, particularly drought tolerance, by studying how these wild eggplant species can survive and grow vigorously without any human intervention in their natural habitat.

Oxygenic photosynthesis plays a crucial role not only as the primary source of our food, fibre, and many valuable substances, but also as a vital dependency for almost all life on Earth [11]. Photosystems I and II are important electron-transfer components that aid in the conversion of absorbed solar energy to chemical energy during photosynthesis [12–14]. Photosystem II efficiency is the most important factor that contributes to the photosynthesis process. Biomass accumulation is a key process resulting from the proper growth and development of plants. The most essential approach for understanding plant physiological processes and gaining quick insights into PSII efficiency is through chlorophyll fluorescence kinetics [12]. It aids in studying core photosynthesis mechanisms, plant responses to environmental change and genetic variations among species [15]. Under extreme stress, the lower photosynthetic rate was attributed to stomatal closure and decreased mesophyll conductivity [16, 17]. Maxwell and Johnson [18] highlighted that chlorophyll fluorescence is a primary indicator of plant tolerance to environmental stresses and that the damage these stresses inflict on the photosynthetic system. Moreover, Hanachi et al. [19] identified eggplant cultivars based on the QY_max of PSII for salt tolerance and Rane et al. [14] for desiccation tolerance in fruit crops, suggesting that ChlaF is a useful tool for identifying alterations in response to abiotic stresses.

Light energy acquired by chlorophyll molecules in a leaf can cause a range of responses; it is used for photosynthesis or can be lost as heat or re-emitted in the form of light, which is known as ChlaF [18]. ChlaF can reveal changes in a plant’s photosynthetic health state. As a result, we chose to use this imaging technique to investigate the genetic variations in the desiccation tolerance of PSII among eggplant and its wild relatives. Conventional non-imaging fluorescence measurements fail to capture spatial heterogeneity within a leaf for ChlaF parameters [20]. Noninvasive assessment of PSII activity through ChlaF is a widely employed technique, and the adaptability of PSII activity to various abiotic factors has established this method as a pivotal tool, offering insights not only into photosynthetic health but also as a comprehensive indicator of plant responses to environmental perturbations [15, 21]. Therefore, employing noninvasive imaging-based techniques for monitoring fluorescence parameters has become essential for obtaining a comprehensive understanding of the factors contributing to this observed heterogeneity [12, 22].

Earlier reports highlighted that eggplant is moderately tolerant to water stress [23] and more drought-resistant than several other vegetables [24]. However, climate change has led to increases in temperature and drought, resulting in a reduction in eggplant yield, which is expected to suffer more in tropical and subtropical regions [25, 26]. Assessment of eggplant wild species germplasm to determine potential sources of tolerance to abiotic stresses, particularly drought, has been carried out based on biochemical traits. Certain wild species exhibit greater tolerance to abiotic stresses than cultivated eggplant, as reported for drought tolerance in *S. elaeagnifolium* [27] and for salinity tolerance in *S. torvum* [28]. Nevertheless, information about abiotic stress tolerance in wild relatives of eggplant accessions is typically limited [4]. In the present study, we hypothesized that the genetic variation in tolerance to desiccation, which often results from exposure to drought, can be explained by variation in photosystem II (PSII) efficiency. To test this hypothesis, we used chlorophyll fluorescence imaging to investigate leaf dehydration tolerance capacity based on photosystem II sensitivity. This study aimed to optimize the protocol for the application of chlorophyll fluorescence technique and to identify promising sources of desiccation tolerance in eggplant wild species.

## Materials and methods

### Experiment location

The present research was carried out at the ICAR-National Institute of Abiotic Stress Management, Baramati in the Pune district of Maharashtra, India. It is located in the agro-ecological region of the Deccan Plateau, which has a hot and semi-arid climate (AER-6) and agro-climatic zone AZ-95, which is Maharashtra’s scarcity zone [29], and receives on an average, 560 mm of annual precipitation, mainly in the form of rainfall.

### Growth conditions and species details

The soil at the experimental site had a pH of 7.4, 0.40% organic carbon, 62.03 kg ha^−1^ total N, 1.45 kg ha^−1^ available P and 71.05 kg ha^−1^ available K. Eggplant wild species were raised in gravelly soil containing 84.68% sand, 8.81% silt and 6.25% clay [30]. During land preparation, 25 t ha^−1^ compost was mixed by tilling the topsoil. The recommended fertilizers doses for eggplant crop production were 150:75:75 kg ha^−1^N:P:K was applied before transplanting the seedlings. Thirty-five-day-old twenty seedlings were transplanted in soil on raised beds. The standard agronomic practices for eggplant were followed after transplanting. The details of the eggplant and its wild relatives used in the present study are given in Table 1. The seeds of the eggplant species were procured from the ICAR-Indian Institute of Horticultural Research, Bengaluru, India.

**Table 1:** Details of the wild eggplant species used in the experiment

Common name	Scientific name	Gene pool
Scarlet eggplant	*Solanum gilo*	GP2
Indian nightshade	*Solanum indicum*	GP3
Brinjal eggplant	*Solanum melongena* L	GP1
Gboma eggplant	*Solanum macrocarpum* L	GP2
Sticky nightshade	*Solanum sisymbriifolium* Lam	GP3
Turkey berry	*Solanum torvum* Sw	GP3

### Collection of leaf samples

The fully opened, 3^rd^ and 4^th^ top leaves were collected from five different plants of each wild eggplant species from the field. Leaves of each species, along with petioles, were harvested at 10 a.m. and immediately placed in a beaker with petioles soaked in distilled water to avoid shock before being moved to a dark environment. Leaf samples were taken in July and August, when the monthly average maximum and minimum temperatures were 30.5 °C and 22.0 °C, respectively. Leaf samples were transported in airtight insulated ice boxes and placed in dark chambers. Following dark adaptation, only the turgid and fresh leaves were selected for desiccation treatments.

### Chlorophyll fluorescence (ChlaF) measurements

**Fig. 1:**
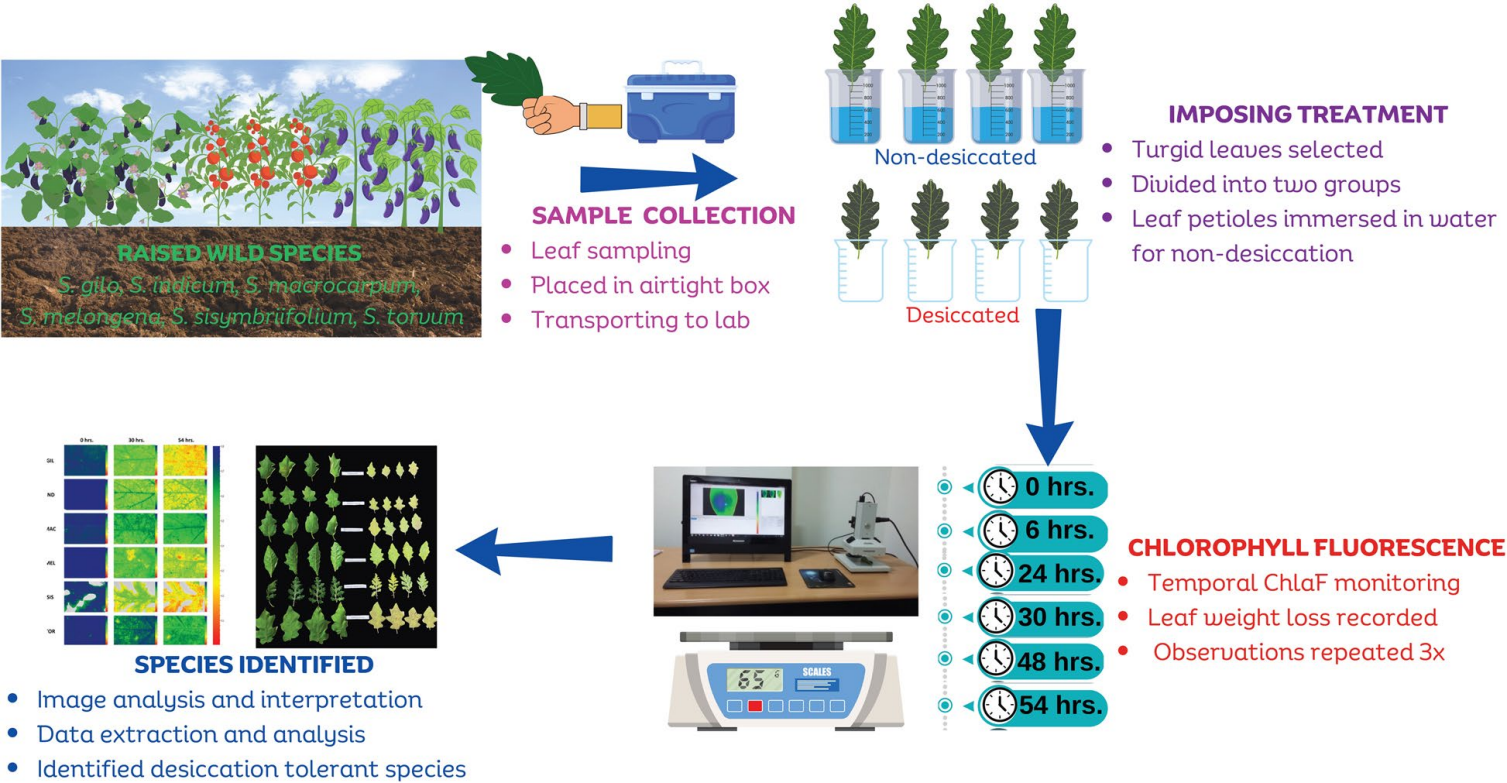
A schematic illustration depicting the experimental arrangement

To measure chlorophyll fluorescence, eight uniform leaves with no signs of withering were chosen. Furthermore, the leaves were divided into two groups: desiccated and non-desiccated (Fig. 1). Each set had four leaves, each of which represents a replicate. The first round of measurement was performed on the leaves in both treatment groups, which were composed of different species, and the readings were taken at 0 h. Subsequently, the petioles of leaves were immersed in distilled water, with care taken to avoid contact with the leaf lamina. Then, leaves from one set of samples were removed from the beakers to apply desiccation treatment, while another set of samples was maintained in beakers with petioles submerged in distilled water to prevent desiccation throughout the experiment. Throughout the experiment, all samples were maintained at room temperature (27 °C) with a 150 µmol (photons) m^−2^ s^−1^. It took approximately 100–120 min to accomplish one set of imaging ChlaF measurements. ChlaF was recorded at 0, 6, 24, 30, 48 and 54-h time points. Similarly, the same set of leaves was monitored continuously for three days, indicating that one set of leaves was desiccated for almost 54 h following leaf detachment.

An image fluorometer (FC 1000-H/GFP, Handy Fluor Cam, PSI, Brno, Czech Republic) was used to quantify ChlaF in leaves at various time points as outlined by [31]. The FluorCam software program was used to detect fluorescence using a high-sensitivity charge-coupled device camera (FluorCam 7). Using non-actinic measurement flashes supplied by super-bright LEDs, images of the dark-adapted fluorescence level (F_0_) were obtained first. To determine the maximal fluorescence (Fm) level, an 800-ms pulse of saturating light radiation [2500 µmol (photons) m^−2^ s^−1^] was applied. The maximum photochemical efficiency of PS-II (QY_max) was computed as follows.

$$\frac{Fv}{Fm}=\frac{(Fm-F0)}{Fm}$$

*F0* minimal fluorescence, *Fm* maximum fluorescence, *Fv* variable fluorescence.

The Fv/Fm pixel value images were represented by a false colour code ranging from red (0.00) to blue (0.80). The difference between Fm and F_0_ is the variable fluorescence (Fv). The Fv and Fm values were then used to determine Fv/Fm, which represents the quantum yield (QY_max) of PSII. We used the ratio of the Fv/Fm of individual leaves under dehydration and saturation conditions to quantify the desiccation response of different eggplant species in terms of the percent loss of the maximum quantum yield of photosystem II (QY_max) as an indication of the loss of fluorescence capability after dehydration. This parameter was then used to estimate the RWC corresponding to a 50 and 75% reduction in QY_max by plotting the best-fit curve of PSII efficiency as a function of loss in relative water content.

### Leaf tissue moisture content and relative water content

The leaf tissue moisture content (TMC) was constantly monitored, and the fresh leaf mass (LM) was measured with a precision balance (BSA423S-CW, Sartorius, Gottingen, Germany) at regular time points immediately before ChlaF imaging. Four observations were recorded under both desiccated and non-desiccated conditions for each species. To estimate the final leaf dry mass, the leaves were dried in a hot air oven at 65 °C for 72 h after the last measurement. The following formula was used to calculate the moisture content of leaf tissue as suggested by Rane et al. [14].

$$TMC\left(\%\right)=\frac{(LM\;at\;the\;time\;of\;measurement-Final\;dry\;mass)}{LM\;at\;the\;beginning\;of\;the\;experiment}\times100$$

The experiment was repeated three times with four replications to confirm the reproducibility of the results. The relative water content (RWC) of dehydrated leaves was calculated using the following formula, with slight modifications, as reported by Trueba et al. [32].

$$RWC\left(\%\right)=\frac{(LM\;of\;dehydrated\;leaf-LM\;of\;dry\;leaf)}{(LM\;at\;the\;beginning\;of\;the\;experiment-LM\;of\;dry\;leaf)}\times100$$

### Histochemical staining assay

Histochemical staining with 3,3-diaminobenzidine (DAB) and nitroblue tetrazolium (NBT) was conducted following the protocol outlined by Juszczak et al. [33], with slight adjustments, to assess leaf damage resulting from hydrogen peroxide (H_2_O_2_) and superoxide anion (O_2_^.−^) production during desiccation stress. After 54 h of desiccation treatment, leaf samples from desiccated and non-desiccated leaves were used for staining. After 54 h, the samples were immediately transferred to the DAB solution (1 mg/mL) for DAB staining. Leaf tissues and DAB solution were placed in a desiccator, subjected to vacuum pressure three times for 5 min each, and then incubated in darkness for 24 h. For NBT staining, leaves were immersed in a 10 mM sodium azide solution, placed in a desiccator, and exposed to 500 psi vacuum pressure for 15 min. Subsequently, the container containing the NBT solution and leaf tissue was subjected to vacuum pressure three times for 5 min each. A destaining solution, composed of glycerol:acetic acid:ethanol in a ratio of 1:1:3 was prepared, and the samples were transferred to it.

### Stomatal density

Fresh leaves were collected from the field and immediately placed in plastic bags. In the laboratory, a thin layer from the abaxial leaf surface was peeled off using a sharp razor. The peeled layer was then placed on a glass slide and observed under a light microscope. The stomatal density was calculated using number of stomata per mm^2^ of leaf.

### Statistical analysis

The experimental data were analysed using R statistical software, version 4.3.2 (R Core Team, 2021). Prior to conducting the statistical analysis, a normality check of the experimental data was performed using the Shapiro–Wilk normality test. Analysis of variance (ANOVA) was carried out in a factorial CRD, through the “Doebioresearch” and “agricolae” packages. Post-hoc mean comparisons were conducted using the least-significant difference (LSD) test (*p* ≤ 0.01). Furthermore, a line curve was generated for assessing the difference between the trends of change in different ChlaF parameters as influenced by time and tissue moisture content using the geome smooth function of ‘ggplot2’. The values presented in the line curve are the means of the replicated treatments.

## Results and discussion

In the present study, a significant variation in photosystem II (PSII) sensitivity in response to the tissue moisture content was evident among wild eggplant species. Initially, we confirmed the effect of the desiccation treatments on the moisture content of the leaf tissue. Subsequently, we examined the effect of tissue moisture level and species on PSII sensitivity in different eggplant species. PSII function was then determined by analysing four distinct parameters obtained from chlorophyll fluorescence (ChlaF) and comparing the threshold of PSII sensitivity for leaf tissue moisture among the species.

### Desiccation effect on tissue moisture content in leaves

**Fig. 2:**
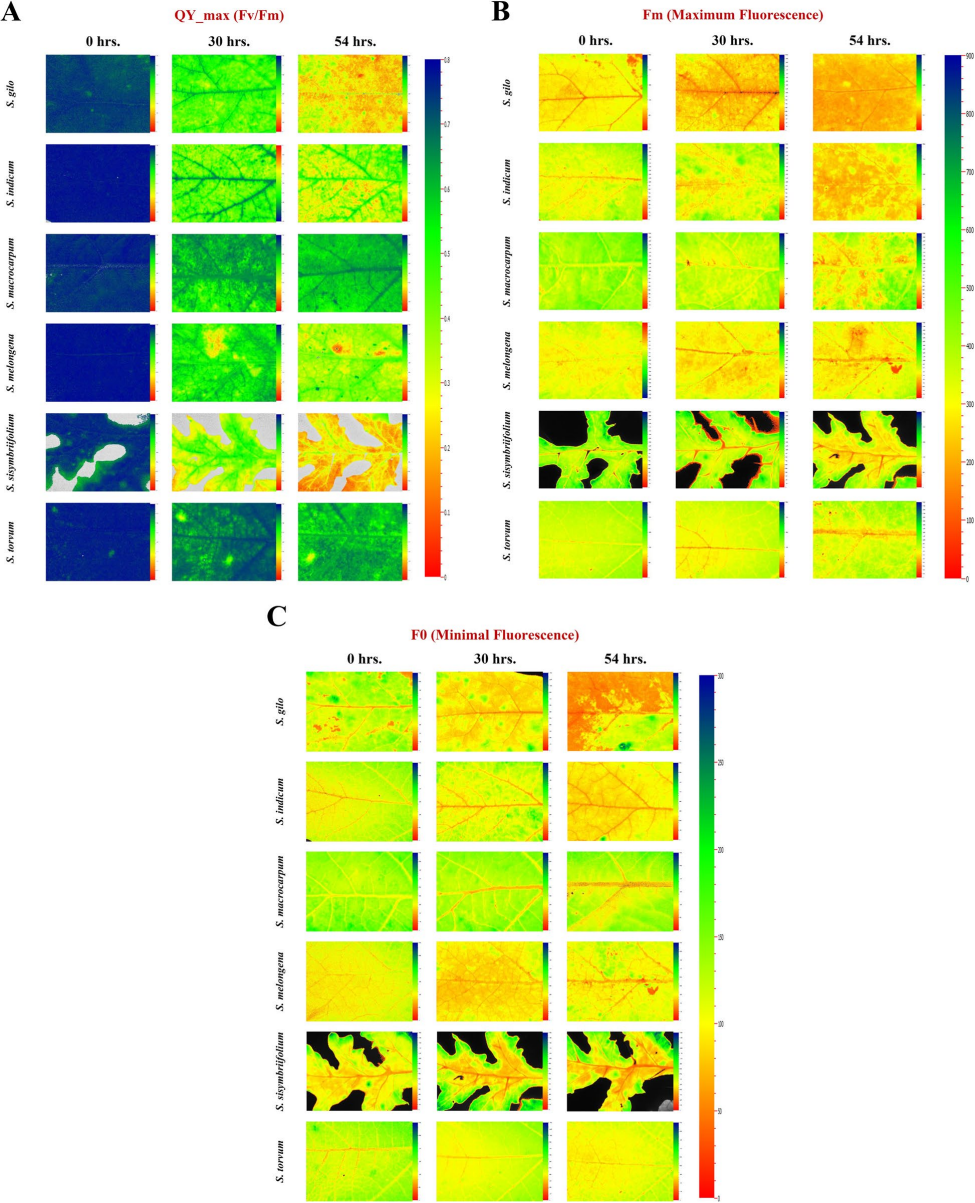
**A** Maximum quantum efficiency (QY_max) images of PSII through ChlaF presented at 0, 30, and 54 h time points after desiccation treatment of representative leaf samples of eggplant species. **B** Maximum fluorescence (Fm) images presented at 0, 30, and 54 h time points after desiccation treatment of representative leaf samples of eggplant species. **C** Minimal fluorescence (F0) images in a dark-adapted leaf presented at 0, 30, and 54 h time points after desiccation treatment of representative leaf samples of eggplant species

**Fig. 3:**
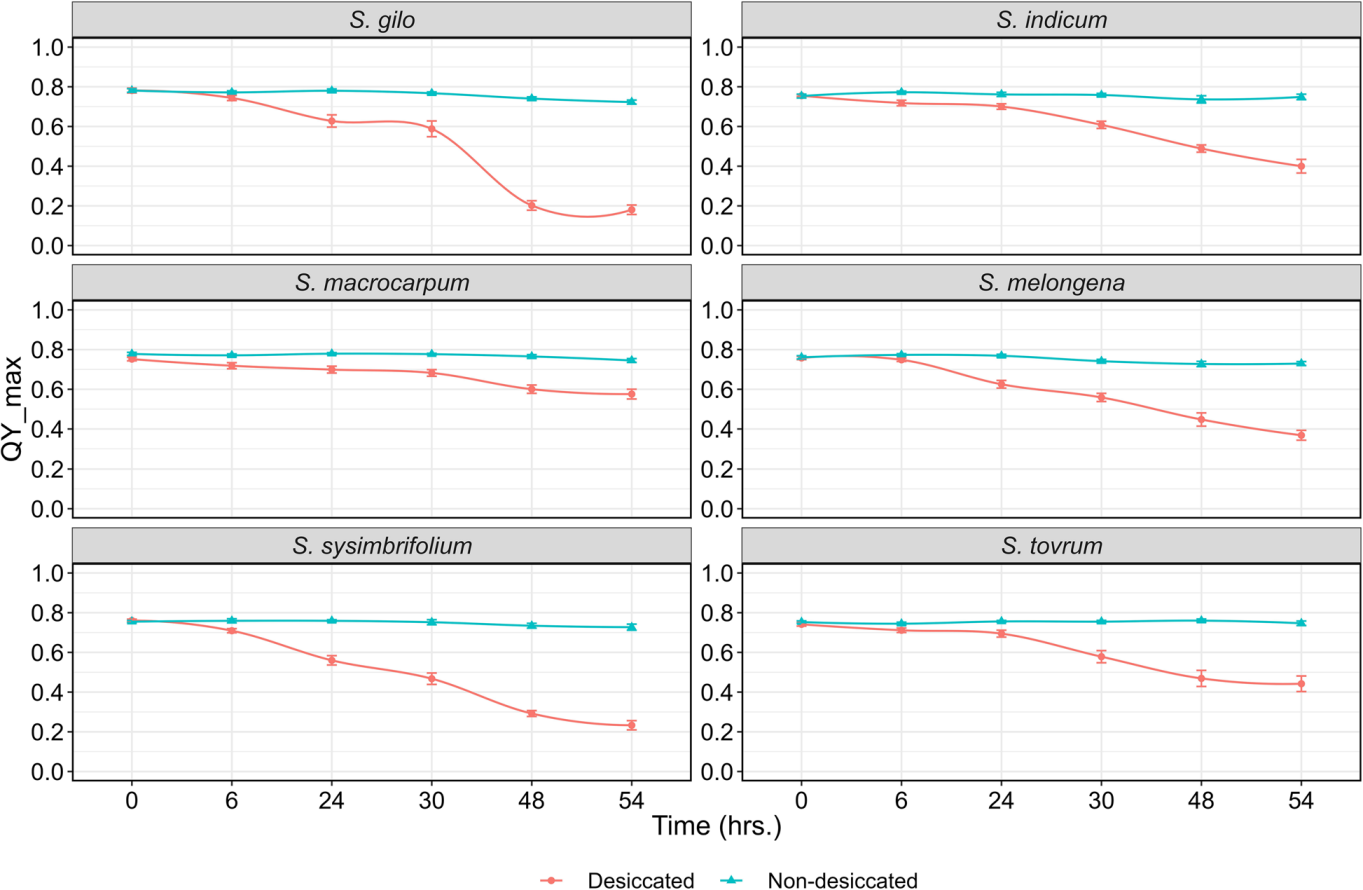
Line curve depicting the temporal effect on QY_max in desiccated and non-desiccated leaves of wild eggplant species. Each data point along the line curve represents the mean of four observations at a given time point, with small vertical bars indicating the SEm

**Fig. 4:**
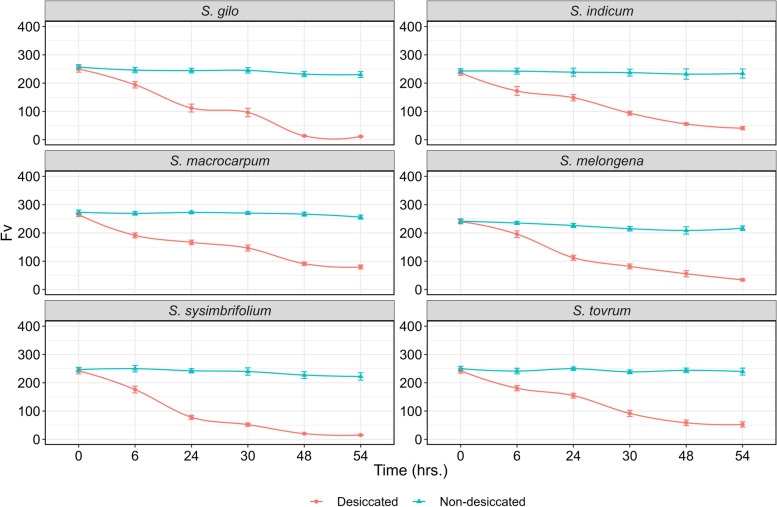
Line curve depicting the temporal effect on Fv in desiccated and non-desiccated leaves of wild eggplant species. Each data point along the line curve represents the mean of four observations at a given time point, with small vertical bars indicating the SEm

**Fig. 5:**
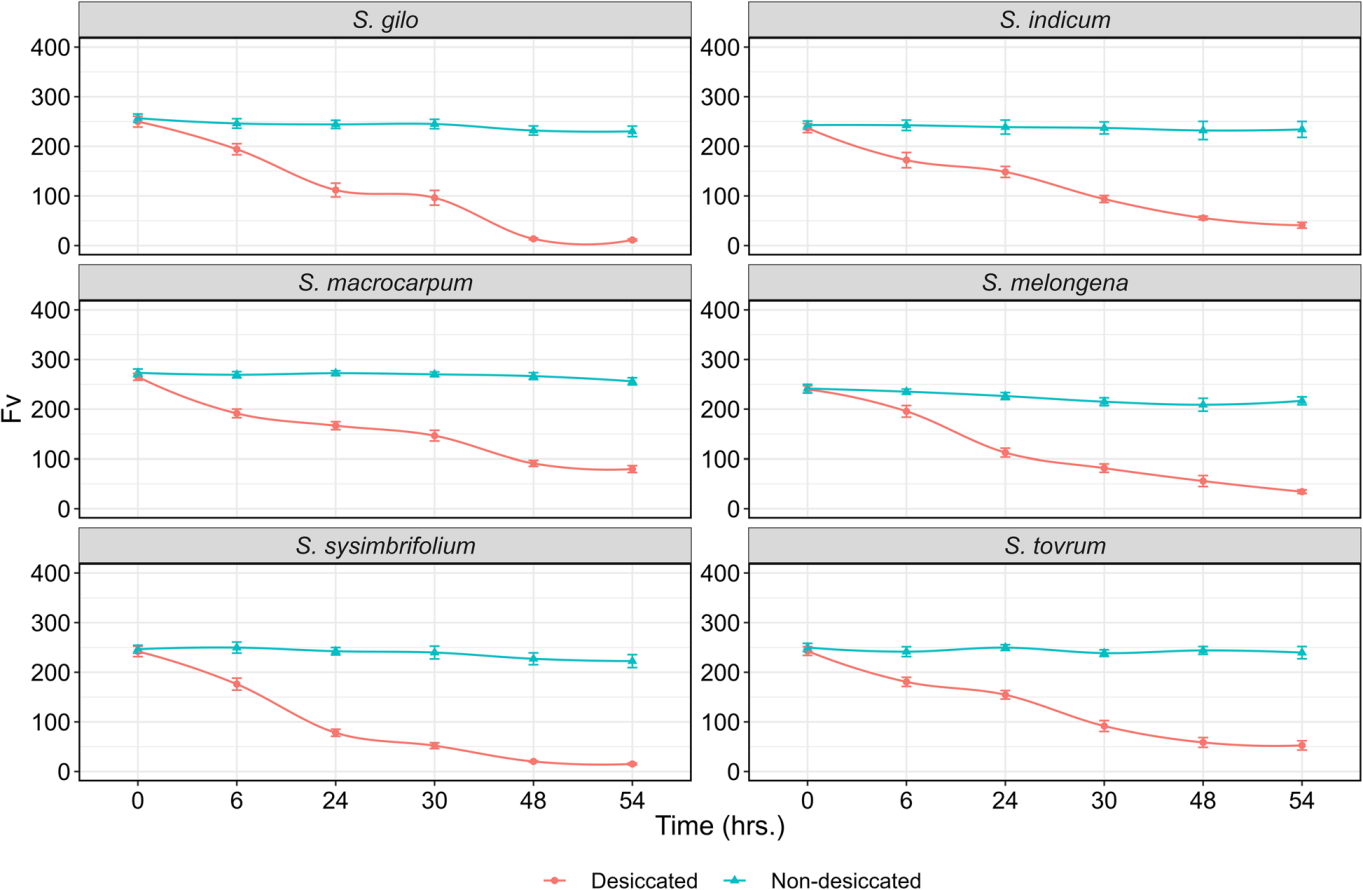
Line curve depicting the temporal effect on Fm in desiccated and non-desiccated leaves of wild eggplant species. Each data point along the line curve represents the mean of four observations at a given time point, with small vertical bars indicating the SEm

Desiccation significantly altered the tissue moisture content (TMC) in the leaves of cultivated and wild eggplant species (Table 2). The TMC strongly influenced the ChlaF parameters and the maximum quantum yield of PSII (Table 3). As there was variation among the species in TMC at a specific desiccation time points, grouping was performed to gain deeper insights into the relationship between QY_max and TMC. We examined maximal fluorescence (Fm), minimal fluorescence (F0), variable fluorescence (Fv), and maximum quantum efficiency of PSII under dark adaptation (Fv/Fm), commonly referred to as QY_max. These parameters are commonly used in abiotic stress screening [15, 34, 35]; hence, these parameters were chosen to evaluate the sensitivity of PSII to desiccation stress in wild eggplant species. F0 represents the minimal fluorescence when all the antenna pigment complexes linked to the photosystem are presumed to be open under dark-adapted conditions. As the duration of leaf desiccation increased, there was a notable decrease in fluorescence parameters, indicating a reduction in photosynthetic efficiency (Fig. 2 A-C). After 54 h of desiccation, F0 and Fm in *S. gilo*, *S. sisymbriifolium*, and *S. melongena* drastically decreased, indicating greater sensitivity to drying. In contrast, *S. macrocarpum*, *S. torvum*, and *S. indicum* displayed the least reduction in fluorescence parameters, suggesting better tolerance to desiccation stress. (Figs. 3, 4 and 5). Our observations align with previous findings, where F0 also decreased in soybean genotypes under drought stress [36] and in citrus rootstocks exposed to drought and flooding [37]. However, a decrease in Fm under high temperature and moisture stress has been reported in previous studies [38, 39].

**Table 2:** Analysis of variance for ChlaF parameters in leaf tissue of eggplant species

Source of variation	DF	F0	Fm	Fv	QY_max
Species	5	1098.6	39,512	28,365	0.27877
TMC	5	5971.8	576,928	465,945	1.87027
Species: TMC	25	93.8	3336	3247	0.06425

*DF* degrees of freedom, *TMC* tissue moisture content, *F0* minimal fluorescence, *Fm* maximum fluorescence, *Fv* variable fluorescence, *QY_max* maximum quantum efficiency of PSII

**Table 3:** Effect of leaf tissue moisture content on ChlaF parameters in eggplant species

TMC (%)	F0	Fm	Fv	QY_max
81 − 90	77.91a	323.90a	245.99a	0.76a
71 − 80	68.47b	253.48b	185.00b	0.72b
61 − 70	63.27c	191.96c	128.69c	0.65c
51 − 60	59.60d	153.29d	93.69d	0.58d
41 − 50	55.24e	104.42e	49.18e	0.42e
31 − 40	53.48e	92.54f	39.05e	0.37f
*P* ≤ *0.001*	***	***	***	***

*TMC* tissue moisture content, *F0* minimal fluorescence, *Fm* maximum fluorescence, *Fv* variable fluorescence, *QY_max* maximum quantum efficiency of PSII. According to the LSD test, the mean values of three replicates followed by the same letter for each factor within each column are not significantly different at *P* ≤ 0.001

At 0 h, the TMC ranged from 81–90%, and the QY_max was 0.76 (Table 3). After 54 h of continuous monitoring, TMC significantly decreased to 31–40%, which was accompanied by a reduction in PSII’s QY_max to 0.37, representing a *ca.* 50% reduction. QY_max (Fv/Fm), a measure of the maximum photochemical efficiency of PSII, is sensitive to drought. The QY_max of plants subjected to prolonged drought stress significantly decreased, indicating a photoinhibition phenomenon [15]. When plants experience abiotic stress, the PSII complex, which is essential for photosynthesis, is often damaged first. This triggers a repair process within the plant, mainly focused on the de novo synthesis of proteins. Notably, the D1 subunit, encoded by the chloroplast gene *psbA*, is a key target for this repair mechanism [40–42]. This repair mechanism is activated in response to photoinhibition, and is commonly initiated by PSII deactivation through photooxidation. The repair process involves the partial disassembly of PSII. The damaged D1 protein is then replaced through de novo synthesis, followed by reassembly of the complete PSII complex [15, 40, 43]. Therefore, despite leaf desiccation in wild eggplant species, higher PSII efficiency may be linked to less photoinhibition and/or a more effective repair mechanism for PSII damage.

A minimal reduction in quantum yield at a lower relative water content may serve as an indicator of the plant’s resilience to desiccation [32]. Our observations are in agreement with these findings, showing similar trends across wild eggplant species subjected to gradual desiccation. Conspicuously, *S. macrocarpum*, *S. torvum* and *S. indicum* were least affected by desiccation, as evidenced by the sustained quantum yield of PSII reaching 61–70% TMC and which then decreased gradually to 31 − 40%. According to the hydraulic-failure theory, a decrease in soil water availability and a high demand for evapotranspiration will result in cavities in xylem conduits and the rhizosphere, which will obstruct water flow and dry plant tissues [44]. Our research focused on the desiccation aspect of drought tolerance because such a phenomenon is extremely important for vegetable crops such as eggplant cultivated with a limited water supply in semiarid regions. During the domestication of crop plants, traits associated with abiotic stress tolerance were less prioritized, resulting in increased vulnerability to abiotic stressors accelerated by climate change [4, 45]. Conversely, wild relatives of eggplant grow naturally in unmanaged landscapes, as they possess traits that enable them to survive with limited water during the post-monsoon or drought period in arid and semiarid regions.

### Temporal effect of treatment on PSII sensitivity

At the beginning of the desiccation treatment (0 h), there was no discernible difference in PSII sensitivity among the species; however, as the duration of desiccation increased to 54 h, notable differences were observed. In fact, the differences in the Fv/Fm ratio among the species became apparent after 30 h of desiccation treatment. After 54 h of desiccation, *S. macrocarpum*, *S. torvum* and *S. indicum* were able to maintain better PSII efficiency than the other species. The robustness of Fv/Fm as a plant health indicator is well-established; healthy photosynthetic tissues typically exhibit a mean Fv/Fm above 0.8. Our initial observations (0 h) reflect this established value, which tended to decline as desiccation progressed (Fig. 2A). Conversely, lower values of Fv/Fm in *S. sisymbriifolium*, *S. gilo* and *S. melongena* at 54 h of desiccation indicated a compromised physiological status, as noted in previous studies [46, 47].

At lower water contents, the effect of stress on PSII photochemistry was more pronounced than the perceptible impact on stomatal conductance and turgor loss [48]. Furthermore, there was declines in Fv/Fm were observed only following the complete loss of midrib xylem hydraulic function in sunflower, as indicated by Cardoso et al. [49]. In our investigation, we observed a remarkable variation in ChlaF parameters from 0 to 54 h of desiccation (Figs. 3, 4 and 5). Furthermore, at 54 h of desiccation, particularly in *S. sisymbriifolium* and *S. gilo*, there was a distinct increase in spatial heterogeneity in photosynthetic performance (QY_max) in the leaf lamina as well as in the mid-rib. Conversely, in tolerant species, spatial heterogeneity in the QY_max of PSII was least evident (Figs. 2A and 3). Similar patterns of spatial heterogeneity in the fluorescence parameters such as minimal ChlaF (F0) and maximum ChlaF (Fm) were detected among the species (Figs. 4 and 5). In a related context, Trueba et al. [32] reported that the impacts of dehydration-induced losses on the rehydration capacity of ten diverse angiosperm species resulted in significant irreversible damage to the photochemical apparatus subjected to extreme dehydration, resulted to full stomatal closure.

### Variations in PSII sensitivity among wild eggplant species

 There were no notable differences in PSII efficiency between the eggplant species during the initiation of desiccation treatment. With the progression of desiccation duration from 0 to 54 h, the differences in PSII efficiency among the species became increasingly conspicuous, particularly after 30 h of desiccation (Fig. 3). The data presented in Table 4 demonstrate a clear difference in ChlaF parameters between desiccated and non-desiccated treatments in eggplant species. Overall, when averaged over desiccated and non-desiccated leaves, *S. macrocarpum* exhibited the highest mean PSII efficiency, followed by *S. indicum* and *S. torvum* (Table 4). The PSII efficiency of *S. macrocarpum* was approximately 1.38-, 2.83-, and 2.22-fold greater than that of *S. melongena*, *S. gilo*, and *S. sisymbriifolium*, respectively. At 54 h under non-desiccated conditions, no variation in PSII efficiency was noted among the species, whereas at its desiccation counterpart, complete inhibition of PSII efficiency was observed in *S. sisymbriifolium* and *S. gilo*, while *S. macrocarpum* was able to maintain distinctly superior PSII efficiency followed by *S. torvum* (Table 4). The Fv reflects the dynamic response of PSII to drought stress. A lower Fv suggests reduced PSII efficiency, whereas a lower Fm provides insight into the overall health and integrity of PSII. A decrease in Fm may indicate stress-induced damage [49]. Our research revealed that compared to other tolerant species, *S. macrocarpum* exhibits superior PSII under desiccation due to its sustained Fm and Fv levels, which contribute to enhanced PSII functionality (Figs. 4 and 5). Fm and Fv contribute to evaluating a plant’s drought tolerance, providing valuable insights for crop breeding and stress-resistant variety selection [50, 51]. Similarly, Rane et al. [14] observed significant variation in tissue dehydration among different dryland fruit crops belonging to various genera. The inter-generic and intra-specific variations in tissue desiccation and PSII efficiency can also be ascribed to leaf anatomy and structural frameworks, which optimize photosynthetic function and gas diffusion [52].

**Table 4:** Mean ChlaF parameters of desiccated and non-desiccated leaves of wild eggplant species at 54 h

Treatment	F0	Fv	Fm	QY_max
*S. gilo*	68.16ab	188.91 cd	120.75d	0.45e
*S. indicum*	65.78b	203.27bc	137.5bc	0.58b
*S. macrocarpum*	71.38a	239.37a	167.98a	0.63a
*S. melongena*	66.86b	189.90 cd	125.55 cd	0.55c
*S. sisymbriifolium*	64.60b	183.45d	118.85d	0.48d
*S. torvum*	68.11ab	214.16b	146.05b	0.59b
*Significance* (S)	*	***	***	***
Desiccated	53.49b	92.54b	39.05b	0.36b
Non-desiccated	81.48a	313.81a	233.17a	0.74a
*Significance* (D)	***	***	***	***
*S. gilo*:Desiccated	48.77d	60.09f	11.32e	0.18f
*S. indicum*:Desiccated	55.71c	96.63e	40.91d	0.40d
*S. macrocarpum*:Desiccated	55.75c	135.66d	79.91c	0.51b
*S. melongena*:Desiccated	55.61c	89.97e	34.36de	0.37d
*S. sisymbriifolium*:Desiccated	48.35d	63.59f	15.24e	0.23e
*S. torvum*:Desiccated	56.75c	109.33e	52.58d	0.44c
*S. gilo*:Non-desiccated	87.56a	317.73b	230.17b	0.72a
*S. indicum*:Non-desiccated	75.84b	309.92bc	234.08ab	0.75a
*S. macrocarpum*:Non-desiccated	87.02a	343.08a	256.06a	0.75a
*S. melongena*:Non-desiccated	78.12b	289.84c	216.75b	0.73a
*S. sisymbriifolium*:Non-desiccated	80.85b	303.32bc	222.47b	0.73a
*S. torvum*:Non-desiccated	79.48b	319.00ab	239.52ab	0.75a
*Significance* (S x D)	***	NS	*	***

*F0* minimal fluorescence, *Fm* maximum fluorescence, *Fv* variable fluorescence, *QY_max* maximum quantum efficiency. The mean values of three replicates followed by the same letter for each factor within each column are not significantly different according to LSD. *NS* non-significant; * and *** indicate significance at *p* ≤ 0.05 and 0.001, respectively

**Fig. 6:**
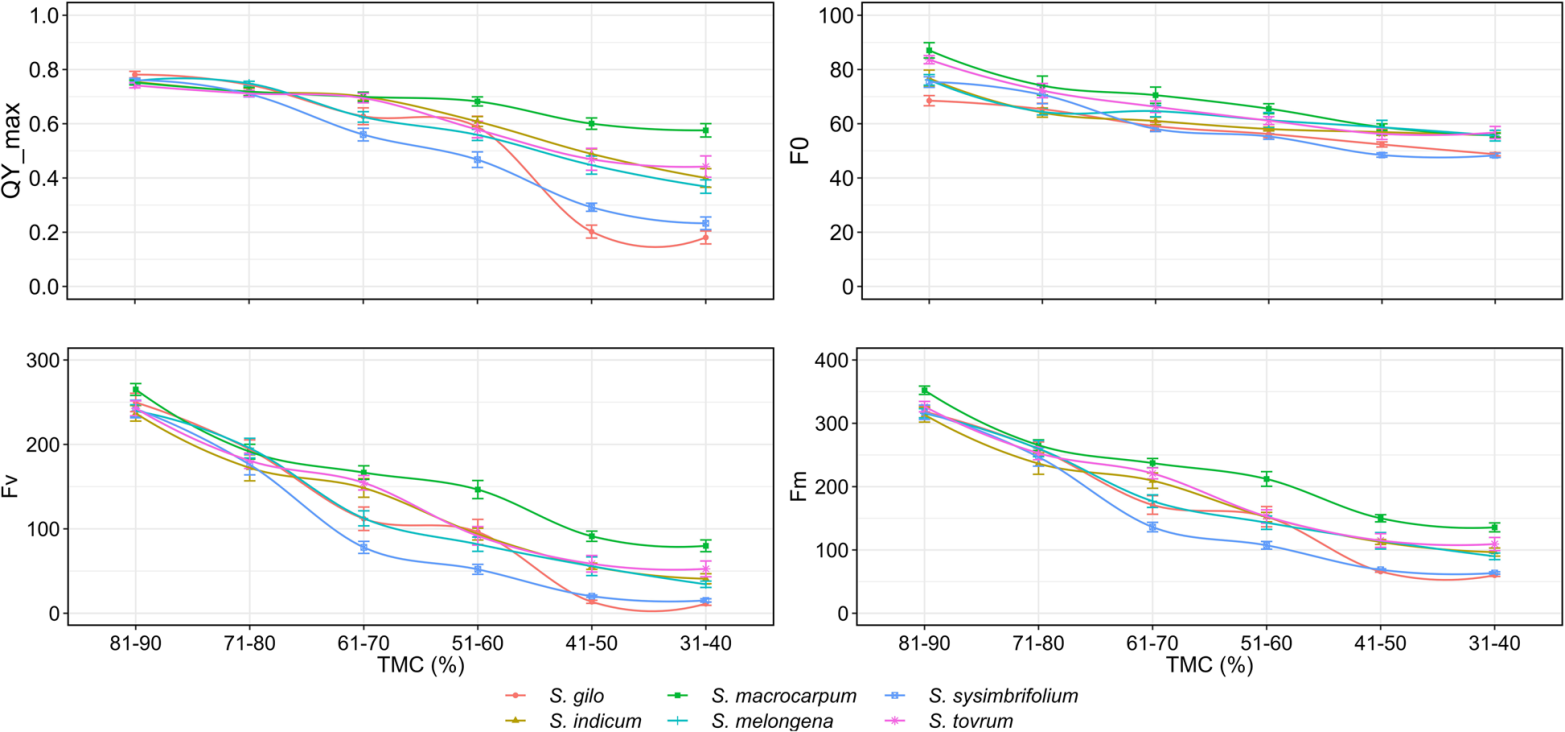
ChlaF dynamics in reaction to leaf desiccation (TMC: tissue moisture content, %), revealed through various parameters (QY_max, F0, Fv and Fm), across leaves of wild eggplant species at varying desiccation levels

A decrease in the QY_max efficiency to less than 0.72 indicates photoinhibition [53]. Our investigation revealed that *S. macrocarpum*, *S. torvum*, and *S. indicum* retained these threshold values of 0.70 up to 60% TMC, unlike other species, which are susceptible to desiccation. Strikingly, among all species, *S. macrocarpum* exhibited desiccation tolerance even up to 50% of that of TMC (Fig. 6). Leaf traits such as trichomes, glaucousness, and epicuticular wax collectively constitute the primary defense mechanism against abiotic and biotic stresses in higher plants [54, 55]. The higher retention of PSII efficiency and desiccation tolerance in *S. macrocarpum* can also be attributed to its glossy and thick leaves, which may act as a barrier to moisture loss. Although *S. sisymbriifolium* is a highly vigorous species, the expression of the PSII efficiency trait in its leaves implies that the mechanism for drought tolerance does not depend upon the vigour of the plant and may be influenced by other traits, considering that drought tolerance is polygenic in nature [56].

Moreover, we hypothesized that greater PSII efficiency in tolerant species could be attributed to their better ability to maintain cellular homeostasis while neutralizing the overproduction of reactive oxygen species (ROS) under desiccated conditions, thereby contributing to improved plant health. Hanachi et al. [19] evaluated four commercial eggplant cultivars under salinity stress and noted that PSII efficiency was unaffected in the Bonica and Galine cultivars due to the undisturbed electron flow chain. This suggests that genetic variations in PSII efficiency exist even at the varietal level within the same species. Under abiotic stresses, such as salinity and drought, photochemical quenching can contribute to protecting the photosynthetic apparatus by transferring electrons to O_2_ [57], resulting in less photoinhibition and a better repair mechanism. This can be attributed to the fact that *S. macrocarpum* and *S. torvum* can effectively manage water loss by continuing an appropriate electron flow chain during periods of desiccation up to 31 − 40% TMC (Fig. 6). Additionally, wild species such as *S. torvum* also exhibit more level of antioxidative defense mechanisms than other species [26]. In addition to improved PSII function in *S. torvum*, antioxidative defense mechanisms might have aided in scavenging reactive oxygen species generated during leaf desiccation.

### Retention of PSII function

**Fig. 7:**
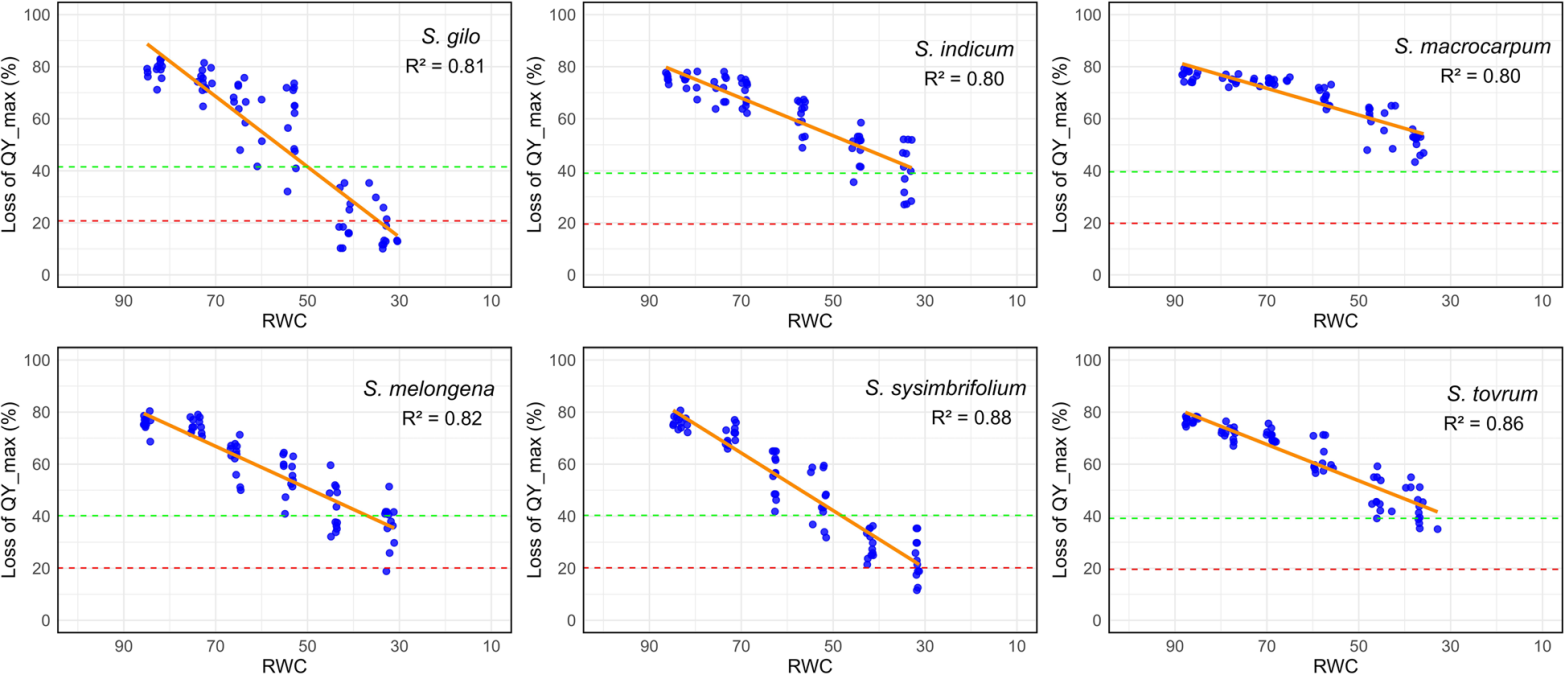
Illustrates the impact of desiccation on the loss of the QY_max of PSII with decreasing leaf relative water content (RWC) in wild eggplant species. The blue dots denote measurements recorded across various samples, while solid regression lines in orange represent fitted models. Additionally, dashed lines indicate the loss of QY_max at 50% (green) and 75% (red) of the initial efficiency. R-squared values were considered significant at a level of *P* ≤ 0.01

The results clearly demonstrated that the loss of PSII function was notably slow in *S. macrocarpum* and *S. torvum*, with *S. indicum* exhibiting comparable retention in desiccated leaves (Fig. 7). Conversely, a drastic decrease in quantum yield was observed for *S. gilo*, followed by *S. sisymbriifolium* and *S. melongena*. The reduction in QY_max efficiency to 50% of the initial value occurred when the relative water content (RWC) reached 50% for *S. gilo* and *S. sisymbriifolium*, and 55% for *S. melongena*. However, the QY_max of *S. macrocarpum* did not decrease to 50% of initial, while for *S. torvum* and *S. indicum*, a 50% loss occurred at 31% and 33% RWC, respectively. *S. macrocarpum* and *S. torvum*, belonging to GP2 and GP3, respectively, exhibited greater tolerance to abiotic stress than cultivated species from GP1. Interestingly, despite belonging to GP3, the highly sensitive *S. sisymbriifolium* showed diversity in the PSII trait among species from the same gene pool but was susceptible to leaf desiccation [4, 26]. Additionally, desiccation-tolerant species that retain greater PSII activity may be able to recover quickly after drought. Although drought tolerance is a polygenic trait, mechanisms beyond PSII efficiency may govern tolerance in these species [35, 56].

To further investigate this phenomenon, we examined stomatal density on the leaves of each species using light microscopy (Supplementary Fig. 1). We observed that *S. torvum*, *S. macrocarpum*, and *S. indicum* exhibited greater desiccation tolerance and PSII efficiency than *S. gilo*, *S. melongena*, and *S. sisymbriifolium*. This difference might be attributed to the first group having lower stomatal density on their leaves, which helps them conserve water. These results are in line with those of Franks et al. [58], who reported that a reduction in stomatal density reduces stomatal conductance by enhancing water use efficiency without hindering photosynthetic ability. Studies on barley have shown that having fewer stomata allows them to use water more efficiently without affecting grain yield [59]. Interestingly, a decrease in stomatal density enhances dehydration tolerance, drought tolerance, and water use efficiency in Arabidopsis, rice, maize, and tomato [60–62]. These findings indicate that stomatal density is a promising target trait for screening a large set of germplasms for breeding programs aiming to develop crop varieties with improved water use efficiency.

### Histochemical staining of leaves

**Fig. 8:**
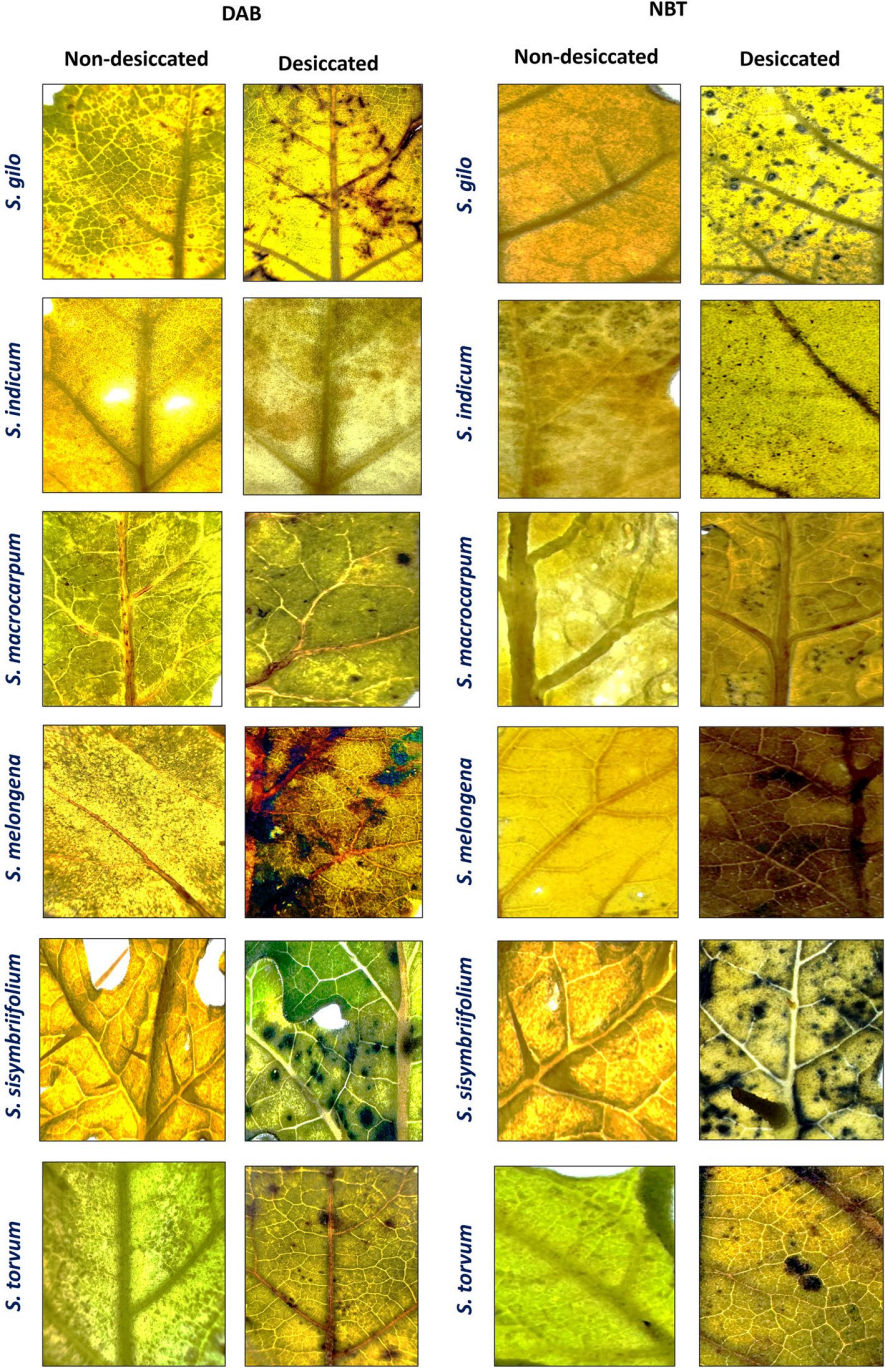
The 3,3-diaminobenzidine (DAB) and nitroblue tetrazolium (NBT) histochemical staining of desiccated and non-desiccated leaves of wild eggplant species

Moreover, after 54 h of desiccation, we conducted histochemical staining on the leaves of wild eggplant species to detect ROS accumulation. Extreme desiccation, whether induced by water or salt stress, can trigger apoptosis through ROS generation. In desiccated photosynthetic tissues, chlorophyll molecules remain excited while carbon fixation is hindered by water scarcity. Under these circumstances, electron flow persists, leading to the transfer of excitation energy from photoexcited chlorophyll pigments to ground-state oxygen (^3^O_2_), resulting in the formation of singlet oxygen (^1^O_2_). Furthermore, damage in photosystem II can produce superoxide (O_2_^.−^), hydrogen peroxide (H_2_O_2_), and the highly toxic hydroxyl radical (OH·) [63]. ROS are generated as part of regular metabolism in the electron transport chains of respiration and photosynthesis; however, their production is drastically increased during desiccation [64]. Similarly, the results of DAB and NBT staining of desiccated and non-desiccated leaf samples of different species clearly revealed differences in the generation of ROS. The desiccation-tolerant species *S. macrocarpum*, *S. torvum*, and *S. indicum* accumulated relatively less ROS (O_2_^.−^ and H_2_O_2_) compared to desiccation-susceptible species (Fig. 8). This result strengthens our argument about the correlation between desiccation tolerance and the generation of ROS.

Based on our findings, *S. macrocarpum* exhibited the greatest desiccation tolerance as assessed through ChlaF parameters, followed by *S. torvum* and *S. indicum*. In contrast, S*. melongena* showed a moderate level of tolerance. Conversely, *S. gilo*, followed by *S. sisymbriifolium*, demonstrated the lowest tolerance to desiccation. Our study revealed variations in desiccation tolerance among wild eggplant species based on the quantum efficiency of PSII through ChlaF dynamics. Since drought is a polygenic and complex trait, other mechanisms of tolerance may exist that need exploration at the genetic level. Therefore, there is scope to identify genes and regulatory pathways associated with desiccation tolerance through genetic and transcriptomic studies. Desiccation-tolerant species identified could be potential sources for breeding climate-resilient eggplant cultivars. Moreover, they can be used as rootstocks for enhancing drought tolerance in commercial elite cultivars through grafting.

## Conclusion

In conclusion, for the temporal monitoring of ChlaF parameters over time, QY_max emerged as the most sensitive parameter for detecting subtle changes in PSII sensitivity due to leaf desiccation in eggplant and its wild relatives. Furthermore, the variations in QY_max among wild eggplant species, along with the greater PSII efficiency observed in the leaves of *S. macrocarpum*, *S. torvum*, and *S. indicum*, emphasize the desiccation tolerance of these species. This highlights a clear decline in traits associated with drought tolerance throughout the domestication of cultivated eggplant, particularly in comparison with its wild relatives, notably those from GP2 and GP3, which exhibit remarkable desiccation tolerance. Consequently, tolerant species hold promise as potential donors for enhancing crop varieties to cope with climate adaptation and resistance to abiotic stresses. To the best of our knowledge, this study represents the first instance of utilizing chlorophyll fluorescence (ChlaF) for evaluating desiccation tolerance during the screening of wild eggplants. The use of imaging-based ChlaF has proven to be a powerful tool for discerning both temporal and spatial variations in leaf photosynthetic performance, facilitating rapid phenotyping of large quantities of crop wild relatives and germplasm to pinpoint traits linked to abiotic stress.

### Supplementary Information

Supplementary Material 1.

## Data Availability

The dataset supporting the conclusions of this article is included within the article.
