# Supplementary figures and images for: Deciphering desiccation tolerance in wild eggplant species: insights from chlorophyll fluorescence dynamics
Source: BMC Plant Biol. 2024 Jul 25;24:702. doi: 10.1186/s12870-024-05430-9 (PMC11270916; doi:10.1186/s12870-024-05430-9)

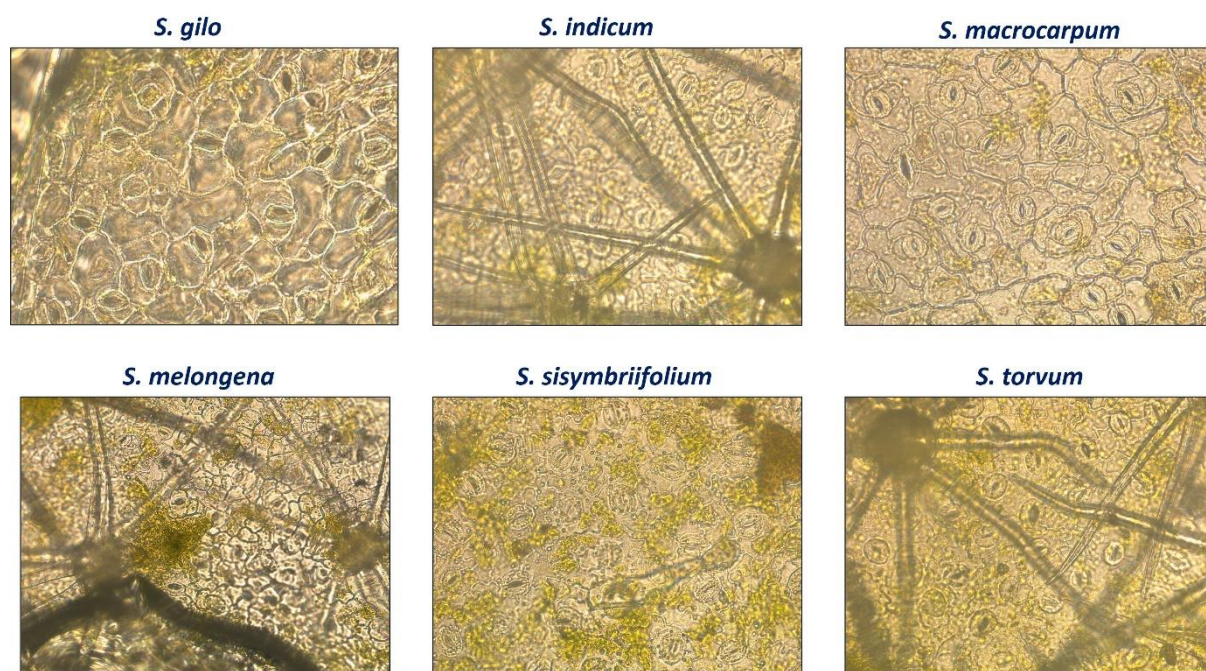

**Supplementary Fig. 1.** Microscopic images of stomatal density of different eggplant species.

Supplement: Supplementary file 1 — Supplementary Material 1. [file 12870_2024_5430_MOESM1_ESM.pdf]
